# Genetically-Driven Enhancement of Dopaminergic Transmission Affects Moral Acceptability in Females but Not in Males: A Pilot Study

**DOI:** 10.3389/fnbeh.2017.00156

**Published:** 2017-08-29

**Authors:** Silvia Pellegrini, Sara Palumbo, Caterina Iofrida, Erika Melissari, Giuseppina Rota, Veronica Mariotti, Teresa Anastasio, Andrea Manfrinati, Rino Rumiati, Lorella Lotto, Michela Sarlo, Pietro Pietrini

**Affiliations:** ^1^Department of Experimental and Clinical Medicine, University of Pisa Pisa, Italy; ^2^Department of Surgical, Medical, Molecular Pathology and Critical Care, University of Pisa Pisa, Italy; ^3^Department of Pharmacy, University of Pisa Pisa, Italy; ^4^Clinical Psychology Branch, Azienda Ospedaliero-Universitaria Pisana Pisa, Italy; ^5^Applied Research Division for Cognitive and Psychological Science, European Institute of Oncology Milan, Italy; ^6^Department of Developmental Psychology and Socialization and Center for Cognitive Neuroscience, University of Padova Padova, Italy; ^7^Department of General Psychology and Center for Cognitive Neuroscience, University of Padova Padova, Italy; ^8^IMT School for Advanced Studies Lucca, Italy

**Keywords:** dopamine, genetic variant, moral behavior, decision-making, moral dilemma

## Abstract

Moral behavior has been a key topic of debate for philosophy and psychology for a long time. In recent years, thanks to the development of novel methodologies in cognitive sciences, the question of how we make moral choices has expanded to the study of neurobiological correlates that subtend the mental processes involved in moral behavior. For instance, *in vivo* brain imaging studies have shown that distinct patterns of brain neural activity, associated with emotional response and cognitive processes, are involved in moral judgment. Moreover, while it is well-known that responses to the same moral dilemmas differ across individuals, to what extent this variability may be rooted in genetics still remains to be understood. As dopamine is a key modulator of neural processes underlying executive functions, we questioned whether genetic polymorphisms associated with decision-making and dopaminergic neurotransmission modulation would contribute to the observed variability in moral judgment. To this aim, we genotyped five genetic variants of the dopaminergic pathway [rs1800955 in the *dopamine receptor D4 (DRD4)* gene, DRD4 48 bp variable number of tandem repeat (VNTR), *solute carrier family 6 member 3* (*SLC6A3*) 40 bp VNTR, rs4680 in the *catechol-O-methyl transferase (COMT)* gene, and rs1800497 in the *ankyrin repeat and kinase domain containing 1 (ANKK1)* gene] in 200 subjects, who were requested to answer 56 moral dilemmas. As these variants are all located in genes belonging to the dopaminergic pathway, they were combined in multilocus genetic profiles for the association analysis. While no individual variant showed any significant effects on moral dilemma responses, the multilocus genetic profile analysis revealed a significant gender-specific influence on human moral acceptability. Specifically, those genotype combinations that improve dopaminergic signaling selectively increased moral acceptability in females, by making their responses to moral dilemmas more similar to those provided by males. As females usually give more emotionally-based answers and engage the “emotional brain” more than males, our results, though preliminary and therefore in need of replication in independent samples, suggest that this increase in dopamine availability enhances the cognitive and reduces the emotional components of moral decision-making in females, thus favoring a more rationally-driven decision process.

## Introduction

Morality and moral judgments are crucial for human social interactions. Since the early days, moral behavior has been a matter of intense philosophical debate. Psychology has mostly focused on the study of the mental processes that subtend the complexity of moral behavior (Osman and Wiegmann, 2017). Over the last decades, the developments of novel methodologies for the *in vivo* study of the brain morphological and functional architecture in a non-invasive manner in humans (Pietrini, 2003; Poldrack, 2012; Poldrack and Yarkoni, 2016), along with the enormous acquisitions from molecular biology and genetics that led to the decoding of the human genome (Venter et al., 2001), have prompted cognitive sciences to venture into the study of the neurobiological mechanisms that subtend mental processes involved in moral behavior. In this perspective, a few brain-imaging studies have investigated brain neural activity in individuals who were asked to make moral choices in regard to distinct scenarios (Greene et al., 2001, 2004; Hutcherson et al., 2015). In their pioneer work, Greene and colleagues have proposed a “dual process theory” of moral decision-making, according to which both cognition and emotion are involved in moral judgments (Greene et al., 2001, 2004, 2008, 2009; Shenhav and Greene, 2014). These authors identified distinct neural patterns associated with emotion and cognition, and suggested that a conflict between these two components occurs during moral judgment formulation. The dual process theory has received additional support by independent studies (Schaich Borg et al., 2006; Valdesolo and DeSteno, 2006; Ciaramelli et al., 2007; Koenigs et al., 2007; Bartels, 2008; Fumagalli et al., 2010a). Moreover, some authors showed that pro-social emotions including aversive emotional reactions to harmful scenarios are highly variable among individuals (Moll and de Oliveira-Souza, 2007; Decety and Cowell, 2014a,b). Similarly, responses to moral dilemmas differ among individuals as well (Sarlo et al., 2014; Rota et al., 2016). The mechanisms that underlie this variability still remain to be understood. Distinct genetic profiles may likely be involved, as different polymorphisms have been associated with definite aspects of behavior including violent and antisocial behaviors (Rigoni et al., 2010; Sartori et al., 2011; Buades-Rotger and Gallardo-Pujol, 2014; Iofrida et al., 2014).

Dopamine is known to affect several aspects of social behavior that are fundamental for moral choices (i.e., motivation, reward, and reinforcing learning). The 7-repeat allele of a polymorphic region within the third exon of the Dopamine Receptor D4 gene (*DRD4*), for example, has been linked to impaired altruistic behavior (Bachner-Melman et al., 2005; Anacker et al., 2013) and decreased empathy (Uzefovsky et al., 2014), both powerful enhancers of pro-social behavior (Eisenberg, 2000, 2007).

These findings consistently suggest that gene variants in the dopaminergic pathway may affect moral decision-making, a crucial function in human sociality. To investigate this hypothesis, we combined a moral judgment paradigm with genetic testing, so to assess the potential role in moral choices of five genetic variants that affect dopaminergic neurotransmission: rs1800955 in the *dopamine receptor D4* (*DRD4*) gene, the *DRD4* 48 bp variable number of tandem repeat (VNTR), the *solute carrier family 6 member 3* (*SLC6A3*) 40 bp VNTR, rs4680 in the *catechol-O-methyl transferase* (*COMT*) gene, and rs1800497 in the *ankyrin repeat and kinase domain containing 1* (*ANKK1*) gene (Table 1).

**Table 1:** Genotype frequencies and Hardy Weinberg equilibrium statistics for each genetic variant in the whole sample (males plus females) and in the two separate genders.

**Polymorphisms**	**Genotype groupings**	**Genotypes**	**Females (102)**	**Males (98)**	**Whole sample (200)**
rs1800955 *DRD4* C521T	C/C	C/C	0.120	0.125	0.245
	T-allele	T/C	0.230	0.220	0.450
		T/T	0.155	0.150	0.305
	Hardy Weinberg equilibrium	*p* = 0.395	*p* = 0.279	*p* = 0.171	
*DRD4* VNTR 48 bp Exon III	non-7r/non-7r	non-7r/non-7r	0.362	0.304	0.666
	7r-allele	7r/non-7r	0.122	0.152	0.274
		7r/7r	0.035	0.025	0.060
	Hardy Weinberg equilibrium	*p* = 0.078	*p* = 0.664	*p* = 0.741	
*SLC6A3* VNTR 40 bp 3′-UTR	9r-allele	9r/9r	0.055	0.050	0.105
		9r/10r	0.261	0.271	0.532
	10r/10r	10r/10r	0.201	0.162	0.363
	Hardy Weinberg equilibrium	*p* = 0.374	*p* = 0.077	*p* = 0.053	
*rs4680 COMT* G472A Val158Met	A-allele	A/A	0.116	0.111	0.227
		G/A	0.241	0.251	0.492
	G/G	G/G	0.151	0.130	0.281
	Hardy Weinberg equilibrium	*p* = 0.652	*p* = 0.827	*p* = 0.865	
*rs1800497 ANKK1* C2137T Glu713Lys	A2/A2 (C/C)	A2/A2 (C/C)	0.345	0.380	0.725
	A1-allele (T-allele)	A1/A2 (T/C)	0.150	0.105	0.255
		A1/A1 (T/T)	0.015	0.005	0.020
	Hardy Weinberg equilibrium	*p* = 0.904	*p* = 0.733	*p* = 0.843	

*r = repeats*.

Each of these variants has been found individually associated with the modulation of personality traits and cognitive abilities linked to moral behavior.

Specifically, the C-allele of rs1800955 variant increases the *DRD4* transcriptional efficiency (Okuyama et al., 1999) and has been associated with augmented extraversion (Bookman et al., 2002; Eichhammer et al., 2005; Golimbet et al., 2005) and novelty-seeking (Munafò et al., 2008), whereas the T-allele has been associated with attention deficits (Yang et al., 2008).

The *DRD4* VNTR encodes the third intracellular loop of the receptor that interacts with a G_i_ protein with an inhibitory effect on cAMP formation (Van Tol et al., 1991). The 7-repeat allele of this polymorphism affects receptor function by inhibiting the ligand binding and *DRD4* expression (Asghari et al., 1994, 1995; Grady et al., 2003; Borroto-Escuela et al., 2011; Knafo et al., 2011; González et al., 2012). It is known that, upon ligand binding, DRD4 forms a functional heterodimer with DRD2; interestingly, the 7-repeat allele of DRD4 interferes with this dimerization, thus causing a reduction of DRD2 activity as well (Borroto-Escuela et al., 2011; González et al., 2012). The same allele negatively influences altruistic traits (Bachner-Melman et al., 2005) and impairs prefrontal cortex activation and connectivity patterns linked to executive functions (Herrmann et al., 2007; Gilsbach et al., 2012). In particular, DRD4 plays a central role in the synchronization of glutamatergic and GABA-ergic activities and the 7-repeat allele impairs the balance between these two networks by causing a higher suppression of glutamatergic signaling (Zhong et al., 2016).

The *SLC6A3* VNTR modulates the dopamine transporter (DAT1) expression, as the 9-repeat allele decreases DAT-binding capacities and increases dopamine availability (Heinz et al., 2000; VannNess et al., 2005). This variant seems to support decision-making processes under risky situations, reward seeking behavior, and cognitive flexibility (Dreher et al., 2009; Zhong et al., 2009; Mata et al., 2012; Fagundo et al., 2014).

rs4680 affects the enzymatic activity of COMT, as the G/A base change leads to a Val/Met amino acidic change and to a less efficient degradation of dopamine (Chen et al., 2004). Brain imaging studies have shown that the A/A genotype increases prefrontal cortex activation related to cognitive performances, providing additional support to the hypothesis that rs4680 plays a role in moral choices (Egan et al., 2001; Malhotra et al., 2002; Bertolino et al., 2004, 2006; Winterer et al., 2006; Ettinger et al., 2008).

rs1800497, also known as Taq1A, is a tag SNP for some genetic variants located in the *dopamine receptor D2* (*DRD2*) (Zhang et al., 2007). Imaging studies showed that, compared to A2/A2 carriers, the A1-carriers have a significant reduction in the number of DRD2-binding sites in the caudate nucleus (Noble et al., 1991; Ritchie and Noble, 2003) and in the striatum (Pohjalainen et al., 1998) and a decreased dopaminergic activity (Noble et al., 1991, 1997). This deficiency in dopaminergic system due to the A1-allele has been associated with substance dependency and abuse (Blum et al., 1996; Vereczkei et al., 2013), with lower performance in executive functions (Fossella et al., 2006; Klein et al., 2007), and with poor cognitive flexibility and decision-making abilities (Fagundo et al., 2014; Marinos et al., 2014).

Because these variants are all located in genes that belong to the same pathway, namely the dopaminergic pathway, they should not be considered as acting independently from each other, but rather synergistically. Therefore, we combined them in multilocus genetic profiles—following the example of Nikolova et al. (2011), Stice et al. (2012), Davis et al. (2013), Davis and Loxton (2013), and Kohno et al. (2016)—representative of the overall functional effect of these variants both on the dopaminergic neurotransmission on one hand and on the cognitive processes that underlie moral choices on the other.

## Materials and methods

### Subjects

Two hundred unrelated Caucasian subjects (102 females) of Italian ancestry, aged 23.1 ± 6.6 SD (standard deviation) years (mean age: females 23.5 ± 7.9 SD; males 22.6 ± 4.8 SD; Table 2), were recruited among students at Pisa and Padua Universities. As the genetic variability of the Italian population is not discrete but continuous, and even more so among people from the Italian peninsula (Di Gaetano et al., 2012), the population stratification was considered of no relevant effect in this group of subjects.

**Table 2:** Demographic and descriptive data of response variables to moral dilemmas, in the whole sample (males plus females) and in the two separate genders (as reported in Rota et al., 2016).

**As in Rota et al. (2016)**		**Whole sample**	**Females**	**Males**
Sample size	N	200	102	98
Age	mean	23.06	23.46	22.64
	SD	6.57	7.93	4.79
Freq_Y	mean	0.48	0.42	0.54^***^
	SD	0.37	0.21	0.23
Acceptability	mean	2.62	2.18	3.08^***^
	SD	1.59	1.27	1.37
(sqrt)RT_Y	mean	99.62	98.69	101.65
	SD	18.99	17.03	13.96
(sqrt)RT_N	mean	97.62	94.71	101.25^*^
	SD	24.68	18.35	17.19
Valence	mean	3.04	2.74	3.38^***^
	SD	1.27	0.88	1.14
Arousal	mean	5.04	4.99	5.09
	SD	1.98	1.93	1.86

0.01 <

* p-value ≤ 0.05;

*** *p-value ≤ 0.001*.

*Data are means ± SD. Significant p-values are highlighted in gray shade*.

None of the subjects reported any history of neurological or psychiatric disorders, as assessed by anamnestic interview conducted by board-certified psychologists. The study was approved by the Local Ethic Committees at both Padua and Pisa Universities. Each participant signed an informed written consent to participate in the study and retained the right to drop out from the study at any moment.

### Experimental paradigm

Participants provided their saliva samples for DNA extraction and answered 56 written moral dilemmas characterized by different types of scenarios, modified from the standardized set of Lotto et al. (2014) (see Supplementary File 1). Each dilemma included a short story that ended by proposing an utilitarian resolution (i.e., the sacrifice of one person to save more people) to the portrayed situation, thus facing the reader with a moral dilemma. Participants read each dilemma at their own pace on a computer screen and indicated whether they would engage in the proposed action by pressing the YES/NO labeled buttons. Labeling of the right and left buttons was counterbalanced across participants. YES answers represented utilitarian responses; for each subject, the frequency of YES answers (Freq_Y) was calculated. Response times for YES (RT_Y) and NO (RT_N) were collected. Furthermore, subjects ranked the moral acceptability (Acceptability) of the proposed actions by using an 8-point Likert-type scale (0 = not at all acceptable, 7 = completely acceptable). The degree of pleasantness in engaging in the proposed actions (Valence) (1 = very unpleasant, 9 = very pleasant) and the extent of emotional activation (Arousal) (1 = not at all, 9 = very much) was evaluated by using the Self-Assessment Manikin (Bradley and Lang, 1994).

### Genotyping

Saliva samples were collected by the ORAGENE•DNA Self-Collection kit OG-500 (DNA Genotek Inc., Kanata, Canada) and DNA was extracted by prepIT•L2P® kit (DNA Genotek Inc., Kanata, Canada), according to the manufacturer's instructions.

rs1800955 and rs1800497 were genotyped by Polymerase Chain Reaction (PCR)-Restriction Fragment Length Polymorphism (RFLP) by using the primers Forward-5′-TCAACTGTGCAACGGGTG-3′/Reverse-5′-GAGAAACCGACAAGGATGGA-3′ (Barr et al., 2001) and Forward-5′-CACGGCTGGCCAAGTTGTCTA-3′/Reverse-5′-CACCTTCCTGAGTGTCATCAA-3′ (Eisenberg et al., 2007), respectively. Digestions were performed with the FastDigest FspI (NsbI) enzyme (Thermo Fisher Scientific Inc., Waltham, MA, USA) and the TaqIα enzyme (New England Biolabs, Ipswich, USA).

*DRD4* VNTR and *SLC6A3* VNTR were genotyped by PCR-Fragment Length Analysis by using the primers Forward-5′-GCGACTACGTGGTCTACTCG-3′/Reverse-5′-AGGACCCTCATGGCCTTG-3′ (Serretti et al., 2006) and Forward-5′-TGTGGTGTAGGGAACGGCCTGAG-3′/Reverse-5′-CTTCCTGGAGGTCACGGCTCAAGG-3′ (Vandenbergh et al., 1992), respectively. PCR products were visualized on agarose gel.

rs4680 was genotyped by PCR-High Resolution Melting (HRM) by using the primers Forward-5′-CAGCGGATGGTGGATTTC-3′/Reverse-5′-TTCCAGGTCTGACAACGG-3′. The HRM analysis was performed with a temperature resolution of 0.2°C ranging from 75°C to 90°C. Data collection and genotype calls were obtained by the Rotor-Gene 6000 series software v1.7 (Qiagen, Venlo, Netherlands) using previously sequenced DNA samples as reference genotypes.

### Statistical analyses

The SPSS Advanced Statistics v21 (IBM Corporation, Armonk, NY, USA) was used to perform the statistical pre-processing and analysis of the collected data.

Deviations from normality of response variables and behavioral scores were evaluated by Kolmogorov-Smirnov and Shapiro-Wilks tests; outlier elimination (below the 5th and above the 95th percentiles) was applied to obtain normalized data. RT_Y and RT_N variables were square root (sqrt)-transformed to normalize their distribution.

In a previously reported behavioral study conducted in the same sample of individuals enrolled for the present research, we found significant associations between the responses to moral dilemmas and personality traits—including impulsivity, venturesomeness, and empathy—and mood states (Rota et al., 2016). Thus, the individual scores from the behavioral scales—the Impulsivity-Venturesomeness-Empathy Questionnaire (I7) (Russo et al., 2011), the Interpersonal Reactivity Index (IRI) (Davis, 1980), and the Profile of Mood State (POMS) (McNair et al., 1971)—were included as covariates in the subsequent genetic association analyses.

Concerning the age of subjects, as it did not correlate with the response variables (Supplementary Table 1), it was not included in the analysis as a covariate.

Deviation from the Hardy-Weinberg equilibrium was evaluated by using the *HardyWeinberg* (Graffelman and Camarena, 2008) and *genetics* (Warnes, 2003) packages in R (www.r-project.org).

The Fisher exact test was used to evaluate group differences in genotype distribution.

To investigate the association between response variables and genotypes in each gender, the Generalized Estimating Equations (GEEs) were used, as they provide an optimal framework to analyze the correlated data that also show different distributions like the adopted variables (Hardin and Hilbe, 2012). Loglinear Poisson or Tweedie with log link function distributions were used to analyze the Freq_Y variable, whereas Gaussian or Tweedie distributions with identity link function were chosen to analyze Acceptability, Valence, Arousal, and sqrt-transformed RT_Y and RT_N variables, as suggested by the goodness of fit values of Quasi Likelihood under Independence Model Criterion (QICC). An exchangeable working matrix appeared to be the most suitable method to model the within-subject dependency.

Multivariate analysis methods based on GEEs are still under development (see Xu et al., 2014) for an example) and no optimal correction method exists to control for multiple comparisons and multiple testing in GEEs. Thus, a Bonferroni correction was applied, though it may be considered even too conservative for interconnected variables, like the selected genetic variants.

First, a single variant analysis was performed to test whether any genetic variant was individually associated to the response variables [Bonferroni correction: (a) analysis in the whole sample: *p* = number of genetic variants (5) × number of response variables (6) = 30; (b) sex by genotype interaction: *p* = number of genetic variants (5) × number of response variables (6) = 30; (c) post hoc: *p* = number of genetic variants (5) × number of response variables (6) × genders (2) = 60]. Then, after excluding any driving effect by any of these single variants, a genetic profile analysis was performed.

Multilocus genetic profiles were created by assigning a score to each homozygous genotype based on the functional effect of the two alleles on dopaminergic signaling (1 = high activity, 0 = low activity). Scores to the heterozygous genotypes were assigned based on scientific literature data describing their combination with one or the other homozygous genotype, in relation to cognitive processes and personality traits associated with moral behavior (see Table 3). Then, for each subject, a global score ranging from 0 to 5 was calculated by counting the number of high activity genotypes. None of the subjects showed an overall count equal to zero or to five.

**Table 3:** Scores assigned to each variant genotype (1 = high activity; 0 = low activity), according to the indicated references, to create the multilocus genetic profiles.

**Polymorphisms**	**Genotypes**	**Multilocus score 0 = Low 1 = High**	**References**
rs1800955 *DRD4* C521T	C/C	1	Okuyama et al., 1999; Ronai et al., 2001; Bookman et al., 2002; Eichhammer et al., 2005; Golimbet et al., 2007; Munafò et al., 2008
	T/C	0	
	T/T	0	
*DRD4* VNTR 48 bp Exon III	non-7r/non-7r	1	Asghari et al., 1994, 1995; Grady et al., 2003; Wang et al., 2004; Borroto-Escuela et al., 2011; Knafo et al., 2011; González et al., 2012
	7r/non-7r	0	
	7r/7r	0	
*SLC6A3* VNTR 40 bp 3′-UTR	9r/9r	1	Heinz et al., 2000; VannNess et al., 2005; Dreher et al., 2009; Forbes et al., 2009; Zhong et al., 2009
	9r/10r	1	
	10r/10r	0	
rs4680 *COMT* G472A Val158Met	A/A	1	Egan et al., 2001; Bertolino et al., 2006; He et al., 2012
	G/A	1	
	G/G	0	
rs1800497 *ANKK1* C2137T Glu713Lys	A2/A2 (C/C)	1	Pohjalainen et al., 1998; Ritchie and Noble, 2003
	A1/A2 (T/C)	0	
	A1/A1 (T/T)	0	

The association analysis was performed both by considering the different multilocus genetic profiles as ordinal variables and by subdividing them into two groups, thus creating a dichotomic variable:

- Multilocus ordinal variable: 1 (18 females and 16 males), 2 (38 females and 44 males), 3 (36 females and 27 males), and 4 (10 females and 11 males) [Bonferroni correction: (a) analysis in the whole sample: *p* = number of response variables (6) = 6; (b) sex by genotype interaction: *p* = number of response variables (6) = 6; (c) post hoc: *p* = number of response variables (6) × genders (2) = 12).
- Multilocus dichotomic variable: Low (scores 1–2) (56 females and 60 males) and High (scores 3–4) (46 females and 38 males) [Bonferroni correction: (a) analysis in the whole sample: *p* = number of response variables (6) = 6; (b) sex by genotype interaction: *p* = number of response variables (6) = 6; (c) post hoc: *p* = number of response variables (6) × genders (2) = 12].

To give additional strength to the results of the multilocus analysis, a multivariate permutation test (10.000 permutations) followed by a Closed Testing procedure (Tippett Step-Down combining function) was run by using the dichotomic variable. The permutation analysis was performed by the Non Parametric Combination based “NPC Test R10” software (Pesarin and Salmaso, 2010).

## Results

Allele and genotype frequencies in our sample were consistent with those reported by 1,000Genome (http://www.1000genomes.org/) and HapMap (http://hapmap.ncbi.nlm.nih.gov/) projects. None of the genotype frequencies deviated from the Hardy-Weinberg equilibrium (see Table 1) and they showed equal distribution in the two genders (Fisher's exact test: *p* = 0.87 for rs1800955; *p* = 0.40 for *DRD4* VNTR; *p* = 0.56 for *SLC6A3* VNTR; *p* = 0.64 for rs4680; *p* = 0.15 for rs1800497).

Descriptive data of response variables to moral dilemmas for each single variant genotype grouping, each multilocus genetic profile (ordinal variable), and each multilocus genetic profile group (dichotomic variable) are summarized in Supplementary Tables 2–4, respectively.

### Single variant association analysis with response variables to dilemmas

No association was detected between the individually analyzed genetic variants and any of the response variables (Bonferroni adjusted *p* > 0.05) (see Supplementary Table 2 for descriptive data).

We observed only an interaction between *DRD4* rs1800955 genotype and gender (Wald chi-square test = 6.785, *df* = 2, p_unadjusted_ = 0.034, p_Bonferroni adjusted_ = 1), as the C/C females, but not the males, rated these actions as more acceptable than the T-allele carriers (C/C females > T-allele females: p_unadjusted_ =0.025, p_Bonferroni adjusted_ = 0.75), and an interaction between gender and rs4680 (Wald chi-square test = 31.567, *df* = 2, p_unadjusted_ = 0.001, p_Bonferroni adjusted_ = 0.03), as female A-allele carriers rated utilitarian choices more acceptable than G/G females (A-allele females > G/G females: p_unadjusted_ = 0.0135, p_Bonferroni adjusted_ = 0.81). Neither one of these *p*-values, however, did survive the Bonferroni correction.

### Multilocus association analysis with response variables to dilemmas

**Figure 1:**
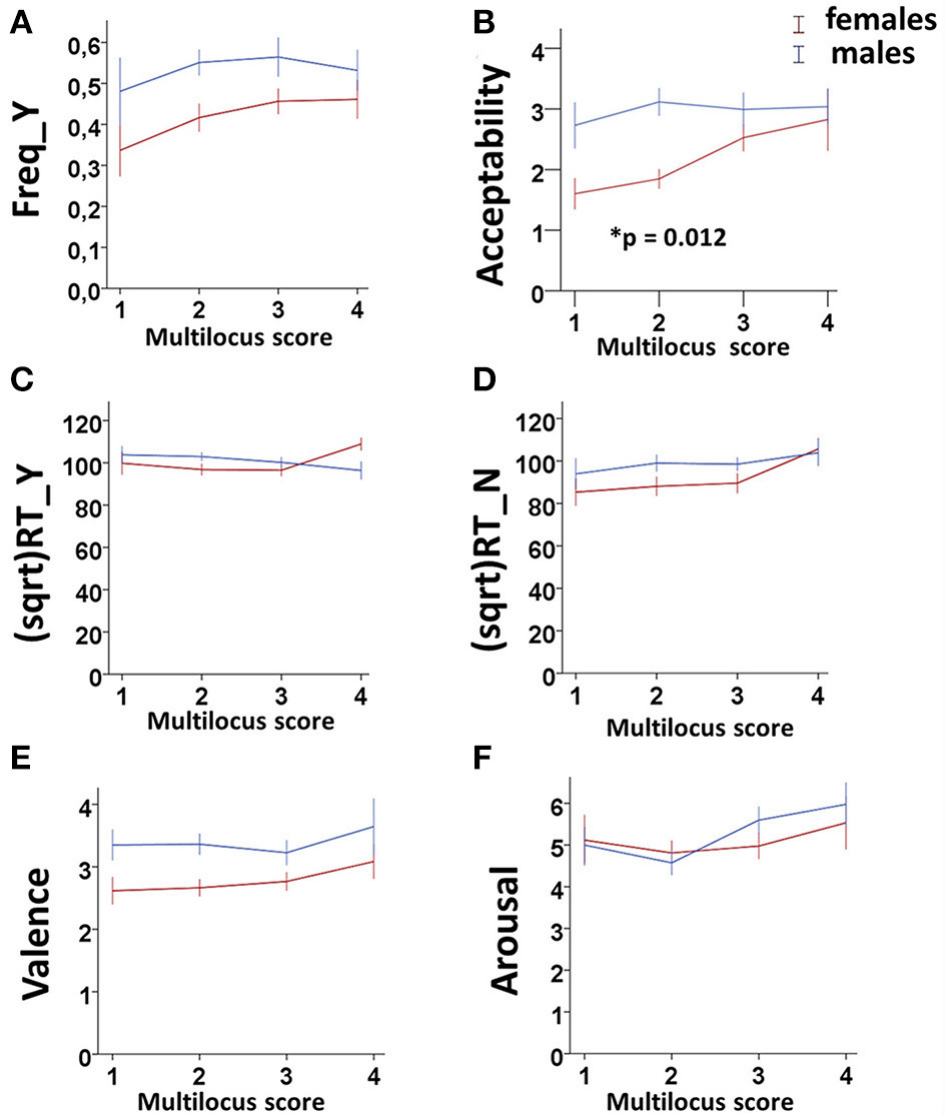
Association results between the dopaminergic Multilocus score and Freq_Y **(A)**, Acceptability **(B)**, (sqrt)RT_Y **(C)**, (sqrt)RT_N **(D)**, Valence **(E)**, and Arousal **(F)** in the two genders. Bars represent mean ± SEM. 0.01 < ^*^p-value ≤ 0.05.

**Figure 2:**
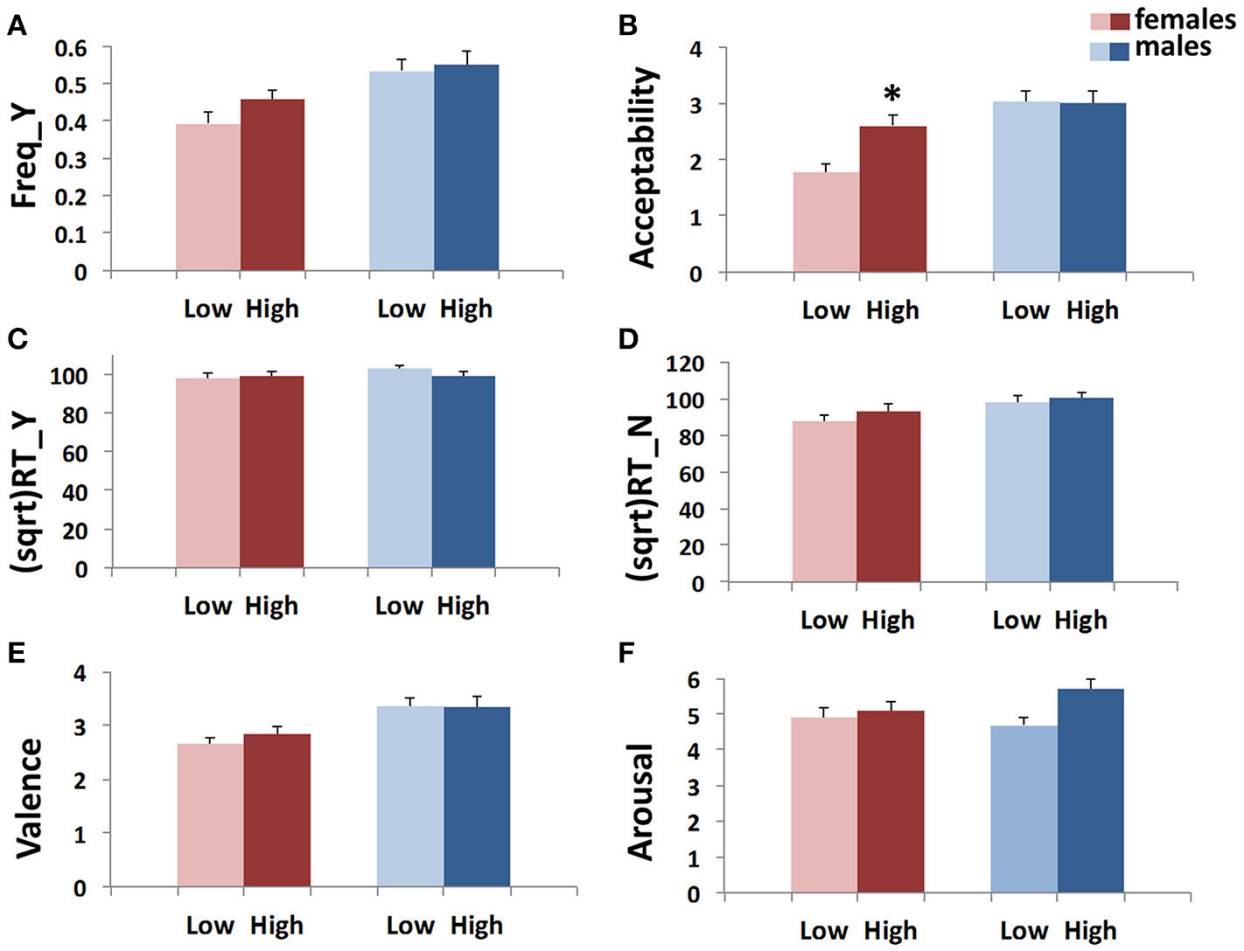
Association results between the dopaminergic dichotomic Multilocus variable and Freq_Y **(A)**, Acceptability **(B)**, (sqrt)RT_Y **(C)**, (sqrt)RT_N **(D)**, Valence **(E)**, and Arousal **(F)** in the two genders. Bars represent mean ± SEM. 0.01 < ^*^p-value ≤ 0.05.

Acceptability:

- **GEE analysis by using the multilocus genetic profiles as an ordinal variable**. No genotype effect was observed when considering the whole sample (males + females). However, an interaction between gender and multilocus genetic profiles was detected (Wald chi-square test = 11.766, *df* = 2, p_unadjusted_ = 0.003, p_Bonferroni adjusted_ = 0.018), as female carriers of High genetic profiles rated utilitarian choices as more acceptable than Low genetic profile females (High females > Low females: p_unadjusted_ = 0.001, p_Bonferroni adjusted_ = 0.012; Figure 1B).
- **GEE analysis by using the multilocus genetic profile as a dichotomic variable**. No genotype effect was observed when considering the whole sample (males + females). However, an interaction between gender and multilocus genetic profiles was detected (Wald chi-square test = 11.597, *df* = 2, p_unadjusted_ = 0.003, p_Bonferroni adjusted_ = 0.018), as female carriers of High genetic profiles rated utilitarian choices as more acceptable than Low genetic profile females (High females > Low females: p_unadjusted_ = 0.001, p_Bonferroni adjusted_ = 0.012; Figure 2B).
- **Multivariate permutation analysis**. The multivariate permutation analysis confirmed the data obtained by GEEs. Overall, a significant effect of multilocus genetic profiles was observed on response variables (Combining function: p_unadjusted_ = 0.007, p_Tippett adjusted_ = 0.019), which survived only in females (Combining function: p_unadjusted_ = 0.004, p_Tippett adjusted_ = 0.012). Specifically, female carriers of High genetic profiles rated utilitarian choices as more acceptable than Low genetic profile females (High females > Low females: p_unadjusted_ = 0.002, p_Tippett adjusted_ = 0.007; Figure 2B).

### Freq_Y, (sqrt)RT_Y, (sqrt)RT_N, valence and arousal

No associations were detected between multilocus genetic profiles and any of these response variables (Figures 1A,C–F, 2A,C–F).

Raw data are reported in Supplementary Data Sheet 1.

## Discussion

In light of the well-known role of dopamine in modulating neural processes associated with executive functions and social cognition, including decision-making (van Schouwenburg et al., 2010; Logue and Gould, 2014; Arnold et al., 2016), reward (Everitt et al., 1999; Schott et al., 2008; Tunbridge et al., 2012), and altruism (Bachner-Melman et al., 2005), the present study tested the hypothesis that genetic variants modulating dopaminergic neurotransmission would affect moral decision-making in healthy individuals. Two hundred individuals were asked to respond to fifty-six moral dilemmas, each one proposing to adopt an utilitarian choice, that is, to sacrifice a person in order to save a larger group of people. Behavioral responses were analyzed in respect to five alleles of genes that regulate dopaminergic neurotransmission and that, taken individually, are known to affect behavioral and personality traits in humans (Balestri et al., 2014; Iofrida et al., 2014; Cherepkova et al., 2016; Heinrich et al., 2016). As the five selected genetic variants were all located in genes belonging to the same biological pathway, they were considered to act synergistically. Thus, after running a single variant analysis that showed no significant association between the single gene variants and the response variables to moral dilemmas, we performed a multilocus analysis following the methodology implemented by other authors (Nikolova et al., 2011; Stice et al., 2012; Davis et al., 2013; Kohno et al., 2016). To this aim, single variant genotypes were combined in multilocus genetic profiles, which are the representatives of the overall effect of different combinations of these alleles both on dopaminergic neurotransmission and on cognitive processes and behavioral traits associated with moral choices.

Interestingly, by applying the multilocus analysis, a gender effect was observed in females carrying genetic profiles that result in a more efficient dopamine signaling due to increased prefrontal dopamine availability (Heinz et al., 2000; Chen et al., 2004), enhanced expression of DRD2 and DRD4 (Pohjalainen et al., 1998; Okuyama et al., 1999; Ritchie and Noble, 2003), or augmentation of cognitive processes (Egan et al., 2001; Bertolino et al., 2006; Gilsbach et al., 2012; Fagundo et al., 2014). These females showed a higher acceptability than females with genetic profiles that impair the dopamine signaling (Figures 1B, 2B). Substantially, females carrying a genetic profile that potentiates the dopamine signaling judged moral dilemmas significantly more acceptable than the other females did, in a way that resembled male behavior.

That moral choices may differ between males and females is a well-known finding (Harenski et al., 2008; Fumagalli et al., 2010a,b; Youssef et al., 2012; Friesdorf et al., 2015). Males usually are more utilitarian than females (Friesdorf et al., 2015). Indeed, in our sample as well, males, as compared to females, opted for the utilitarian choice more frequently, took more time in responding when they opted for the NO answer, and judged the proposed actions more acceptable and less unpleasant (Table 2; Rota et al., 2016).

In addition, our findings are in agreement with results from a study that used a completely different experimental approach (Fumagalli et al., 2010a). These authors observed an increase in utilitarian responses to a moral judgment task in a group of females who underwent anodal transcranial Direct Current Stimulation (tDCS) over their ventral prefrontal cortex (VPC). As dopamine is an anionic catecholamine, the authors hypothesized that the anodal VPC-tDCS increased dopamine levels in the frontal lobe of these individuals, thus influencing their decisional processes. In contrast, anodal VPC-tDCS did not produce any significant effects in males.

Altogether, these findings raise the challenging question of why a further increase in dopamine signaling makes females more similar to males in moral judgment. Women, in fact, have higher levels of dopamine than men in the prefrontal cortex, independently from genotype, as estrogens down-regulate *COMT* gene expression (Xie et al., 1999) and function (Ball et al., 1972). They also have a higher D2-like receptor binding potential (Kaasinen et al., 2001), so that one would expect that a further increase in dopamine availability should amplify, rather than reduce, differences between genders. However, males and hyper-dopaminergic females may stand at the opposite ends of the inverted U-shaped curve that describes the relationship between dopamine levels and cortical function (Vijayraghavan et al., 2007; Cools and D'Esposito, 2011; Avery et al., 2013). As far as *COMT* is concerned, a gene by gender interaction has been reported also to modulate cortical thickness, neuronal density, and working memory performance differently in males and females, both in humans and in mice (Sannino et al., 2015). Specifically, a genetic reduction in COMT enzyme activity increased cortical thickness in the prefrontal and postero-parieto-temporal cortex in males but not in females, increased neuronal density in males whereas reducing it in females, and impaired working memory in females, but not in males (Sannino et al., 2015).

These findings reinforce our observation of a sexual dimorphism of dopaminergic genetic variants and are in line with the assumption of a gender-specific functional organization in the brain. Males and females, for example, are different as far as addiction behavior is concerned and these differences seem to be due to dissimilarities in the neural systems that mediate positive and negative reinforcement, probably modulated by hormones (Bobzean et al., 2014; Barth et al., 2015; Hammerslag and Gulley, 2016). Furthermore, a sexual dimorphism exists for cognition, as the gender differences in cognitive profiles seem to be associated with distinct multivariate patterns of resting-state functional connectivity detected by magnetic resonance imaging (Satterthwaite et al., 2015).

Males and females activate different cortical brain areas during moral tasks (Harenski et al., 2008; Juan Yang and Mingming, 2014). For example, in individuals rating the degree of moral violation in a series of unpleasant pictures, a stronger association between moral ratings and neural activity in posterior cingulate and insula was seen in females, and between moral ratings and neural activity in inferior parietal cortex was seen in males (Harenski et al., 2008). These results are in line with the hypothesis that female moral concerns may be mostly based on empathetic skills, whereas male moral assessment is mainly rational. Indeed, involvement of the posterior cingulate has been observed in response to social moral dilemmas (Robertson et al., 2007), whereas the involvement of the inferior parietal cortex may indicate that males used mostly cognitive resources to complete the moral tasks (Harenski et al., 2008).

On the basis of the results of the present study, we propose that the genetically driven increase in dopamine signaling may enhance specific executive functions in females, including attention and cognitive flexibility (Logue and Gould, 2014), making them more similar to males in approaching moral issues.

To date, only a very few studies have ventured in exploring the genetic correlates of moral choices. Three studies have identified associations between three different polymorphisms in the oxytocin receptor gene and moral judgment (Walter et al., 2012; Bernhard et al., 2016; Shang et al., 2017). Another work, published by Marsh et al. (2011), has shown that a genetic variation within the promoter region of the serotonin transporter gene (5-HTTLPR) has an impact on moral judgment as well. Our findings expand the current knowledge by providing a first indication in support of a gender-specific role for dopamine-related genes in human moral behavior.

## Limitations of the study

The use of a candidate gene approach, with a restrict number of a priori selected genetic alleles, may be considered a limitation of this study. The main concern about candidate gene studies, in fact, is the low rate of data reproducibility (Duncan and Keller, 2011; Dick et al., 2015). However, even Genome Wide Association Studies (GWAS), in which all the most common genetic variants are genotyped simultaneously without the need of making any a priori selection, are not able to overcome the risk of generating artifacts (Flint and Munafò, 2013). As a matter of fact, data from scientific literature suggest that these two approaches are complementary and they are both valid instruments to find genetic associations (Chang et al., 2014). Furthermore, the genetic variants for the present study were selected based on a strong a priori hypothesis.

Also, although our sample size—two hundred subjects—is comparable to that of the Study 1 described by Bernhard et al. (2016) or even larger than those in other published genetic association studies regarding moral dilemmas (Marsh et al., 2011; Walter et al., 2012), it is still relatively small, so that this may limit the statistical power.

However, to increase the statistical power of our sample, we performed a multilocus analysis by combining the different genotypes for each single variant in genetic profiles representative of the overall effect of these variants on dopaminergic transmission. This methodology has been successfully implemented by other authors (Nikolova et al., 2011; Stice et al., 2012; Davis and Loxton, 2013; Davis et al., 2013; Kohno et al., 2016). The main criticism to this approach is represented by the assumption that the effects of the single variants are considered additive rather than epistatic and with similar magnitude. However, compared to the single gene variant analysis, this strategy allows for a better representation of the effect of biological networks on complex phenotypes, as it is the case with human behavior (Saez et al., 2014).

Furthermore, in order to avoid type I errors, we applied a Bonferroni correction to GEE analysis to control for multiple comparisons and for multiple testing.

Finally, a multivariate permutation analysis was conducted in parallel.

Nonetheless, though our investigation was based on a strong a priori hypothesis and data were subjected to a conservative and rigorous statistical procedure, yet it should be considered as a pilot study with original preliminary findings that warrant replication in independent and larger samples.

## Conclusions

Our findings represent the first indication that genetic factors that modulate dopaminergic neurotransmission may exert a gender selective effect on human moral behavior. For the first time to our knowledge, in fact, we showed that genetics affects male and female moral judgment in a different manner. Specifically, we demonstrated that a genetic profile that improves dopaminergic signaling selectively influences moral judgment in females, making their responses to moral dilemmas more similar to those given by males. As females usually provide more emotionally based answers and engage more the ‘emotional brain’ than males do (e.g., Fumagalli et al., 2010a,b), the enhancement in dopamine availability may improve the cognitive and reduce the emotional counterparts of moral reasoning in females, thus favoring more rational choices.

Our findings, though obtained in a relatively small population and therefore in need of replication in independent samples, prompt additional research, including brain imaging studies designed to investigate patterns of brain activity in response to emotional and rational processing associated with moral judgment tasks (Hutcherson et al., 2015), in male and female carriers of the above reported genotype variants.

## Ethics statement

This study was approved by the Local Ethic Committees at both Padua and Pisa Universities. All subjects gave written informed consent in accordance with the Declaration of Helsinki and retained the right to drop out from the study at any moment.

## Author contributions

SPel and SPal equally contributed to the work: specifically, SPel conceived the work and the experimental design; contributed to data analysis; interpreted and discussed the data, and wrote the manuscript; SPal contributed to the experimental design, to genotyping, data interpretation and manuscript writing and performed most of the statistical analysis; CI performed genotyping and contributed to data interpretation; EM contributed to statistical analysis; GR recruited the subjects, administered moral dilemmas and psychometric scales and collected saliva samples at Pisa University; critically reviewed the manuscript; VM contributed to genotyping and data interpretation; critically reviewed the manuscript; TA performed the permutation tests; AM, LL, and MS enrolled subjects, administered moral dilemmas and psychometric scales and collected saliva samples at Padua University; critically reviewed the manuscript; RR contributed to the experimental design and critically reviewed the manuscript; PP conceived the work, contributed to data interpretation and manuscript writing and critically reviewed the final manuscript.

### Conflict of interest statement

The authors declare that the research was conducted in the absence of any commercial or financial relationships that could be construed as a potential conflict of interest.

## Supplementary material

Supplementary Data Sheet 1 Examples of moral dilemmas administered to the subjects enrolled in the study.
