# Supplementary material for: Genetically-Driven Enhancement of Dopaminergic Transmission Affects Moral Acceptability in Females but Not in Males: A Pilot Study
Source: Front Behav Neurosci. 2017 Aug 29;11:156. doi: 10.3389/fnbeh.2017.00156 (PMC5581873; doi:10.3389/fnbeh.2017.00156)
Supplement: Supplementary file 1 [file Table1.PDF]

Supplementary table 1. Association data between response variables to moral dilemmas and age. Statistical data are reported as Wald-chi square test.

Response variables	Intercept	Wald chi-square values	df	p values
**Freq_Y**	Age	1.642	1	0.200
**Acceptability**	Age	0.757	1	0.384
**(sqrt)RT_Y**	Age	0.157	1	0.692
**(sqrt)RT_N**	Age	1.814	1	0.178
**Valence**	Age	0.017	1	0.895
**Arousal**	Age	0.000	1	0.995
