# Supplementary material for: Genetically-Driven Enhancement of Dopaminergic Transmission Affects Moral Acceptability in Females but Not in Males: A Pilot Study
Source: Front Behav Neurosci. 2017 Aug 29;11:156. doi: 10.3389/fnbeh.2017.00156 (PMC5581873; doi:10.3389/fnbeh.2017.00156)
Supplement: Supplementary file 2 [file Table2.pdf]

Supplementary table 2. Descriptive data of response variables to moral dilemmas for each genotype grouping of the five genetic variants in the whole sample (males plus females) and in the two separate genders. Data are means ± SD.

Polymorphisms	Response variables	Genotype groups	Whole sample		Females		Males	
			Means	SD	Means	SD	Means	SD
rs1800955 *DRD4* C521T	Freq_Y	C/C	0.52	0.38	0.44	0.37	0.59	0.38
		T-allele	0.47	0.36	0.42	0.35	0.52	0.36
	Acceptability	C/C	3.08	1.76	2.77	1.88	3.37	1.59
		T-allele	2.47	1.50	2.00	1.28	2.98	1.55
	(sqrt)RT_Y	C/C	100.60	18.80	102.31	19.04	99.07	18.50
		T-allele	99.28	19.06	97.23	19.58	101.22	18.37
	(sqrt)RT_N	C/C	101.59	25.46	101.56	23.62	101.59	27.16
		T-allele	96.35	24.30	92.00	23.71	101.13	24.06
	Valence	C/C	3.10	1.39	2.81	1.24	3.38	1.46
		T-allele	3.02	1.22	2.67	1.03	3.38	1.30
	Arousal	C/C	5.45	2.16	5.54	2.20	5.37	2.12
		T-allele	4.90	1.90	4.82	1.93	4.99	1.30
DRD4 VNTR 48 bp Exon III	Freq_Y	non-7r/non-7r	0.49	0.37	0.42	0.20	0.53	0.23
		7r-allele	0.46	0.37	0.42	0.23	0.57	0.24
	Acceptability	non-7r/non-7r	2.67	1.65	2.16	1.29	2.95	1.41
		7r-allele	2.57	1.52	2.11	1.24	3.14	1.48
	(sqrt)RT_Y	non-7r/non-7r	100.18	18.82	98.46	16.51	102.81	13.25
		7r-allele	99.00	19.19	99.23	18.49	99.28	15.17
	(sqrt)RT_N	non-7r/non-7r	97.56	24.53	91.47	24.29	98.15	26.64
		7r-allele	97.68	24.86	86.99	33.04	99.34	19.10
	Valence	non-7r/non-7r	3.05	1.27	1.71	0.90	3.32	1.21
		7r-allele	3.03	1.26	2.78	0.84	3.42	0.96
	Arousal	non-7r/non-7r	4.99	2.01	4.90	1.85	5.39	1.80
		7r-allele	5.10	1.95	5.22	2.12	4.56	1.90
SLC6A3 VNTR 40 bp 3'-UTR	Freq_Y	10/10	0.46	0.36	0.39	0.33	0.56	0.37
		9-allele	0.49	0.37	0.44	0.37	0.53	0.37
	Acceptability	10/10	2.59	1.57	2.16	1.33	3.11	1.69
		9-allele	2.64	1.60	2.20	1.58	3.06	1.51
	(sqrt)RT_Y	10/10	96.65	19.08	95.63	20.15	97.69	17.92
		9-allele	101.31	18.75	100.27	19.00	102.23	18.50
	(sqrt)RT_N	10/10	94.58	24.26	92.28	24.43	97.61	23.76
		9-allele	99.35	24.76	95.47	23.67	102.99	25.23
	Valence	10/10	3.15	1.35	2.73	1.07	3.67	1.48
		9-allele	2.97	1.21	2.69	1.10	3.24	1.25
	Arousal	10/10	5.07	1.95	4.68	2.04	5.56	1.71
		9-allele	5.02	2.00	5.20	1.98	4.85	2.01
rs4680 *COMT* G472A Val158Met	Freq_Y	A-allele	0.50	0.36	0.45	0.36	0.55	0.37
		G/G	0.42	0.37	0.34	0.34	0.51	0.38
	Acceptability	A-allele	2.63	1.50	2.36	1.47	2.91	1.49
		G/G	2.59	1.80	1.75	1.43	3.57	1.69
	(sqrt)RT_Y	A-allele	99.44	18.52	99.35	18.84	99.52	18.25
		G/G	100.14	20.26	96.32	21.22	103.98	18.54
	(sqrt)RT_N	A-allele	98.30	26.00	96.18	25.73	100.38	26.13
		G/G	95.91	20.94	89.76	19.00	103.77	20.71
	Valence	A-allele	3.09	1.24	2.83	1.10	3.34	1.31
		G/G	2.91	1.33	2.40	1.00	3.51	1.42
	Arousal	A-allele	5.06	1.95	5.12	1.94	4.99	1.96
		G/G	5.00	2.06	4.67	2.17	5.37	1.85
rs1800497 *ANKK1* C2137T Glu713Lys	Freq_Y	A1-allele	0.47	0.37	0.42	0.35	0.52	0.39
		A2/A2	0.48	0.37	0.42	0.36	0.55	0.36
	Acceptability	A1-allele	2.45	1.44	2.00	1.21	2.86	1.52
		A2/A2	2.69	1.64	2.24	1.56	3.17	1.58
	(sqrt)RT_Y	A1-allele	101.39	19.05	98.37	20.96	104.02	16.83
		A2/A2	99.02	18.95	98.57	19.10	99.45	18.81
	(sqrt)RT_N	A1-allele	99.82	23.50	96.22	24.74	103.54	21.59
		A2/A2	96.83	25.05	93.48	23.74	100.43	25.93
	Valence	A1-allele	2.94	1.18	2.60	1.07	3.25	1.19
		A2/A2	3.08	1.29	2.74	1.09	3.44	1.40
	Arousal	A1-allele	4.85	1.98	5.23	1.95	4.50	1.95
		A2/A2	5.11	1.98	4.91	2.03	5.32	1.89
