# Supplementary material for: Genetically-Driven Enhancement of Dopaminergic Transmission Affects Moral Acceptability in Females but Not in Males: A Pilot Study
Source: Front Behav Neurosci. 2017 Aug 29;11:156. doi: 10.3389/fnbeh.2017.00156 (PMC5581873; doi:10.3389/fnbeh.2017.00156)
Supplement: Supplementary file 3 [file Table3.PDF]

Supplementary table 3. Descriptive data of response variables to moral dilemmas in each Multilocus score group in the whole sample (males plus females) and in the two separate genders. Data are means ± SD.

Response variables	Multilocus score	Whole sample		Females		Males	
		Means	SD	Means	SD	Means	SD
Freq_Y	1	0.41	0.29	0.34	0.25	0.48	0.32
	2	0.49	0.22	0.42	0.21	0.55	0.21
	3	0.50	0.22	0.46	0.19	0.56	0.25
	4	0.50	0.16	0.46	0.15	0.53	0.17
Acceptability	1	2.14	1.44	1.55	1.12	2.75	1.52
	2	2.54	1.44	1.86	1.00	3.13	1.52
	3	2.72	1.40	2.52	1.34	2.99	1.45
	4	2.94	1.27	2.83	1.61	3.05	0.93
(sqrt)RT_Y	1	101.86	15.28	99.81	20.57	103.78	16.23
	2	101.16	18.23	96.81	16.69	102.95	13.58
	3	98.38	15.37	96.51	16.81	100.22	13.35
	4	102.34	13.57	108.89	9.68	96.39	14.22
(sqrt)RT_N	1	89.49	27.90	85.32	26.59	93.93	29.42
	2	93.92	27.89	88.05	28.25	98.86	26.86
	3	93.38	24.43	89.54	28.46	98.49	16.90
	4	104.72	18.35	105.66	16.31	103.87	20.79
Valence	1	2.97	0.98	2.62	0.89	3.35	0.96
	2	3.04	1.07	2.66	0.86	3.36	1.14
	3	2.96	0.99	2.77	0.90	3.23	1.06
	4	3.38	1.25	3.08	0.88	3.65	1.50
Arousal	1	5.06	2.01	5.11	2.35	5.00	1.68
	2	4.69	1.89	4.80	1.85	4.57	1.95
	3	5.24	1.81	4.98	1.88	5.60	1.71
	4	5.77	1.85	5.55	2.03	5.98	1.75
