# Supplementary material for: Genetically-Driven Enhancement of Dopaminergic Transmission Affects Moral Acceptability in Females but Not in Males: A Pilot Study
Source: Front Behav Neurosci. 2017 Aug 29;11:156. doi: 10.3389/fnbeh.2017.00156 (PMC5581873; doi:10.3389/fnbeh.2017.00156)
Supplement: Supplementary file 4 [file Table4.PDF]

Supplementary table 4. Descriptive data of response variables to moral dilemmas in High and Low groups of the dichotomic Multilocus variable in the whole sample (males plus females) and in the two separate genders. Data are means ± SD.

Response variables	Dichotomic Multilocus	Whole sample		Females		Males	
		Means	SD	Means	SD	Means	SD
Freq_Y	Low	0.47	0.24	0.39	0.22	0.53	0.24
	High	0.50	0.21	0.46	0.18	0.56	0.23
Acceptability	Low	2.42	1.44	1.77	1.03	3.02	1.51
	High	2.78	1.36	2.59	1.39	3.01	1.31
(sqrt)RT_Y	Low	100.81	16.11	98.13	17.83	103.17	14.18
	High	99.22	14.96	99.32	16.25	99.11	13.53
(sqrt)RT_N	Low	92.80	27.77	87.62	27.46	97.64	27.40
	High	96.21	23.48	93.05	26.99	100.05	17.99
Valence	Low	3.02	1.04	2.65	0.85	3.36	1.09
	High	3.07	1.07	2.83	0.90	3.35	1.19
Arousal	Low	4.79	1.91	4.90	1.97	4.68	1.88
	High	5.37	1.83	5.10	1.90	5.71	1.70
