# Supplementary material for: Genetically-Driven Enhancement of Dopaminergic Transmission Affects Moral Acceptability in Females but Not in Males: A Pilot Study
Source: Front Behav Neurosci. 2017 Aug 29;11:156. doi: 10.3389/fnbeh.2017.00156 (PMC5581873; doi:10.3389/fnbeh.2017.00156)
Supplement: Supplementary file 5 [file Presentation1.PDF]

Supplementary File 1
Exemplary experimental material

. *You are walking on a footbridge spanning the tracks. You notice that a trolley is headed for five workers on the tracks. On a different track there is a single man at work. To prevent the five people from dying, you divert the trolley onto this track. You know that a man will die and that the other five people will survive.*

. *You are a captain of the military Flying clubs. Your soldiers have mistakenly dropped a missile, which is going to hit a house where there is a family of three people. You send a missile to hit it and provoke a change in its trajectory. The missile will hit an area where there has just been a car accident and where a wounded man is waiting for the Red Cross to come. You know that this man will die and that the family of three people will survive.*

. *You are walking on a footbridge spanning the tracks. You notice that a trolley is headed for five workers on the tracks. You are standing next to a fat man. You push this man off the footbridge into the pat of the trolley. You know that this man will die and that the other five workers will survive.*

. *You are on a rubber dinghy with a friend of yours and a stranger. You know that this man is severely ill and that he is going to die in six months. Far away from you notice a boat that has caught fire. Three people who were on board are now about to drown. To save these people you must reach them very quickly. You push the stranger in the water so that the rubber dinghy is lighter and you can reach them. You know that the stranger will die and that the other three people will survive.*

. *You are on a hot-air balloon together with other four people. You just reached 6000 feet as you notice that there is a malfunctioning of the engine, making you loose altitude very quickly. You are approaching the Swiss mountains and you are going to crash if you don't gain altitude quickly. To lighten the weight of the hot-air balloon you push off one of the people that is with you. You know that this person will die and that yourself and the others will survive.*

. *You are climbing a mountain with three people. You are all tied to the same rope and you are the third of the row. You all slip down and you are stuck in suspension above a rift. The body weight of you all is too big. The robe is going to break soon. Thus, you detach the connection that ties yourself to the last buddy on the row, who is severely injured, to make him fall down. You know that this person will die and that yourself and the others will survive.*

. *You are travelling on a highway with three people. All at the sudden the driver loses consciousness and the vehicle starts to speed up. A truck in front of you is slowing down. The crash between your car and the truck is imminent. On the right, you notice a small parking slot where a stranger is standing. To avoid the impact against the truck you grasp the wheel and turn vigorously on the right. You know that the stranger will be run over and will die and that yourself and the other three people will survive.*

. *Your country is at war. The enemies have occupied your village and are searching every house to kill civilians. You and other three people are hiding in a cellar. A severely wounded person of your group loses control and starts to cry loudly. If the soldiers hear the noise they will find you and kill you all. To prevent that, you push your hand on her mouth to smother her. You know that she will die and that yourself and the other two people will survive.*
